# Stress Brings Memories to the Fore

**DOI:** 10.1371/journal.pbio.1001007

**Published:** 2010-12-21

**Authors:** Rachel Jones

**Affiliations:** Freelance Science Writer, Welwyn, Hertfordshire, United Kingdom

**Figure:**
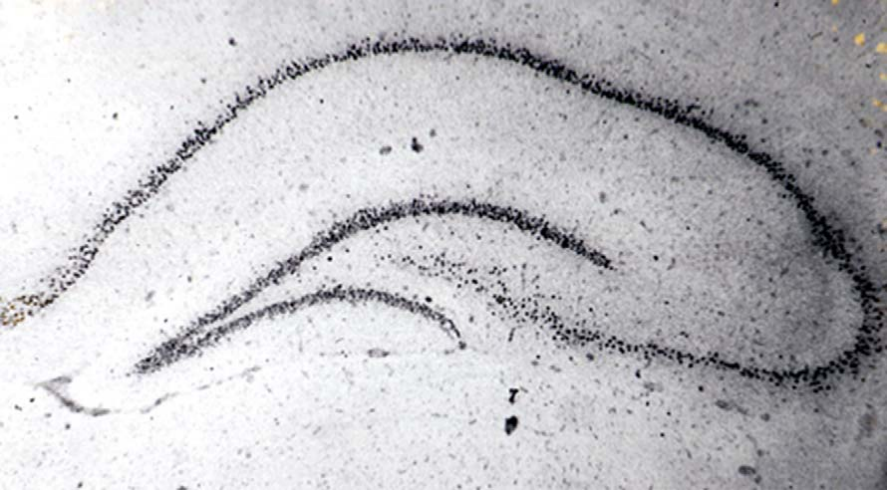
In addition to the well-known role of stress in stabilizing memories, new work demonstrates that stress can also activate and even modify already stable memories, even those that have nothing to do with the stressful experience. The figure shows tritium labeled corticosterone in the rat hippocampus, a brain area crucial for the stress-dependent activation of memory.

A common feature of post-traumatic stress disorder and various mood and anxiety disorders is the formation of negative associations with neutral stimuli, or the recall of negative memories stimulated by unrelated, neutral circumstances. However, the contribution of stress itself to these phenomena is unclear. Now, Fenton and colleagues have shown that in rats stress can reactivate memories that were unrelated to the stressful experience itself, which might contribute to the pathological formation of inappropriate associations in these disorders.

In their initial experiments, the authors trained rats to make a left/right discrimination in a T-shaped maze. One day later, rats were placed into a covered bucket in which they had to swim—an established way of inducing stress in rats. Compared with rats that were placed into shallow water and did not have to swim, the rats that had undergone the stressful swim showed enhanced memory for the T-maze task for at least six days. To ensure that the learning process itself was not a source of stress, the authors tested corticosterone levels in the rats at various points in the experiments and found that corticosterone increased only during the stressful swim procedure.

Stress is known to increase the consolidation of “labile” memories, which are active memories that have not yet been consolidated into long-term memory. Since it is possible that the memory for the initial learning task was still undergoing consolidation one day later at the time of the stressful swim, the authors needed to control for this. Fenton and colleagues used electroconvulsive shock (ECS), which has an amnestic effect on memories that are still undergoing consolidation. Rats that underwent training and then received ECS one day later showed no change in subsequent memory, indicating that the memory for the initial task had completed consolidation at that point. However, rats that received ECS after a stressful swim did not show the stress-induced enhancement of memory that was induced by the swim alone. This result indicates that stress reactivated a stable memory and made it vulnerable to the amnestic effects of ECS.

In another test of the hypothesis that stress reactivates stable memories, the authors induced lateralized memory by training rats in which one hemisphere of the cortex had been temporarily inactivated. These rats showed reduced memory for the training task when they were tested with the contralateral “trained” hemisphere deactivated. However, if they underwent a stressful swim in between the training and testing sessions, they showed interhemispheric transfer of the memory—they were able to recall it using the “untrained” hemisphere, even when the “trained” hemisphere was deactivated. This indicates that the reactivation of the memory by stress allowed it to be transferred between the hemispheres. By contrast, if the trained hemisphere was deactivated during the stressful swim, there was no sign of interhemispheric transfer.

Finally, the authors tested the role of the hippocampus in this phenomenon. Pharmacological deactivation of the hippocampus had no effect on the acquisition or recall of memory in these experiments, but, if the hippocampus was deactivated during the stressful swim, it prevented the stress-induced enhancement of memory. Bilateral inactivation of the hippocampus before the stressful swim also prevented interhemispheric transfer in the lateralized memory protocol.

These results led the authors to hypothesize that stress can reactivate unrelated memories that are stored outside the hippocampus and render them labile through a mechanism that requires the hippocampus. They suggest that, in humans, traumatic stress might reactivate non-traumatic memories and link them to the traumatic memory, thereby facilitating the pathological effects seen in post-traumatic stress disorder and other conditions.

**Ježek K, Lee BB, Kelemen E, McCarthy KM, McEwen BS (2010) Stress-Induced Out-of-Context Activation of Memory. doi: 10.1371/journal.pbio.1000570**

